# Survey on fan-beam computed tomography for radiotherapy: Imaging for dose calculation and delineation

**DOI:** 10.1016/j.phro.2023.100522

**Published:** 2023-12-06

**Authors:** Esther Decabooter, Guido C. Hilgers, Joke De Rouck, Koen Salvo, Jacobus Van Wingerden, Hilde Bosmans, Brent van der Heyden, Sima Qamhiyeh, Chrysi Papalazarou, Robert Kaatee, Geert Pittomvils, Evelien Bogaert

**Affiliations:** a Department of Radiation Oncology (Maastro Clinic), GROW School for Oncology, Maastricht University Medical Centre+, Maastricht, The Netherlands; b Radiotherapiegroep, Arnhem/Deventer, The Netherlands; c Department of Radiotherapy, AZ Sint Lucas, Ghent, Belgium; d Department of Radiotherapy, AZ Sint-Maarten, Mechelen, Belgium; e Department of Medical Physics, Haaglanden Medical Centre, Leidschendam, The Netherlands; f Department of Medical Radiation Physics, University Hospital Leuven, Belgium; g IBiTech-MEDISIP, Department of Electronics and Information Systems, Ghent University, Ghent, Belgium; h Department of Oncology, Laboratory of Experimental Radiotherapy, KU Leuven, Leuven, Belgium; i University Hospitals Leuven, Department of Radiation Oncology, Leuven, Belgium; j Department of Radiotherapy, Leiden University Medical Center, Leiden, The Netherlands; k Radiotherapy Institute Friesland, Leeuwarden, The Netherlands; l Department of Radiation-Oncology, Ghent University Hospital, Ghent, Belgium

**Keywords:** Radiotherapy, Computed tomography, Quality assurance, Survey, Planning CT, Protocol optimization

## Abstract

**Background and purpose:**

To obtain an understanding of current practice, professional needs and future directions in the field of fan-beam CT in RT, a survey was conducted. This work presents the collected information regarding the use of CT imaging for dose calculation and structure delineation.

**Materials and methods:**

An online institutional survey was distributed to medical physics experts employed at Belgian and Dutch radiotherapy institutions to assess the status, challenges, and future directions of QA practices for fan-beam CT. A maximum of 143 questions covered topics such as CT scanner availability, CT scanner specifications, QA protocols, treatment simulation workflow, and radiotherapy dose calculation. Answer forms were collected between 1-Sep-2022 and 10-Oct-2022.

**Results:**

A 66 % response rate was achieved, yielding data on a total of 58 CT scanners. For MV photon therapy, all single-energy CT scans are reconstructed in Hounsfield Units for delineation or dose calculation, and a direct- or stoichiometric method was used to convert CT numbers for dose calculation. Limited use of dual-energy CT is reported for photon (N = 3) and proton dose calculations (N = 1). For brachytherapy, most institutions adopt water-based dose calculation, while approximately 26 % of the institutions take tissue heterogeneity into account. Commissioning and regular QA include eleven tasks, which are performed by two or more professions (29/31) with varying frequencies.

**Conclusions:**

Dual usage of a planning CT limits protocol optimization for both tissue characterization and delineation. DECT has been implemented only gradually. A variation of QA testing frequencies and tests are reported.

## 1 Introduction

Imaging has a key role in radiation therapy (RT). Computer tomography (CT) was reported as the most frequently used imaging modality in a European survey under the auspices of ESTRO-HERO conducted in 28 countries in the period 2009–2014 [1]. According to an IAEA survey, 98 % of the departments globally had access to a fan-beam CT scanner for RT planning, and 70 % had a dedicated fan-beam CT scanner located in the RT department itself [2]. However, existing guidelines on its quality assurance (QA) are relatively old [3], [4], [5], [6] and may have become outdated due to technical advances in the field of CT imaging [7]. Clinical translation of these innovations has increased [8], [9] and in some cases deemed mandatory [10].

To obtain an understanding of current practice, professional needs and future directions in the field of fan-beam CT in RT, a new survey was warranted. The most recent surveys focused on specific use of CT imaging such as respiratory motion management [11], 4DCT [12], stopping power prediction [13] and surface guidance [14]. Due to its broad scope, results are reported in two separate papers. This paper focusses on imaging for dose calculation and delineation. Another paper [15] reports on respiratory motion management, surface guidance as well as future developments.

## 2 Materials and methods

In 2021, the Netherlands Commission on Radiation Dosimetry (Nederlandse Commissie voor Stralingsdosimetrie, NCS) called for a task group to formulate updated guidelines on the QA of fan-beam CT in radiation oncology. An institutional survey was performed by this task group. A maximum of 143 questions covered the following topics: institutions’ and respondent’s information, availability of CT scanners, technical specifications of the CT scanner(s), quality assurance (QA), simulation workflow, imaging for dose calculation, respiratory motion management, surface guided imaging and future vision on fan-beam CT in RT. The actual number could vary since the answers to more generic questions could trigger additional conditional questions. Information on the technical specifications of up to three fan-beam CT scanners could be provided by the respondents.

The survey design was first reviewed for concept validity, term consistency and unambiguity, whereafter it was uploaded to Teams (Microsoft Corp., Redmond, Washington). Subsequently, Belgian and Dutch institutions were invited by email to answer the questions between September 1st and October 10th, 2022. We asked for one completed survey from each of the 47 institutions in Belgium and the Netherlands and provided the option to do this anonymously. The terminology defined in the survey and used throughout this paper includes the abbreviations: MPA (Medical Physics Assistant^3^), MPE RT (Medical Physics Expert Radiotherapy), MPE RD (Medical Physics Expert Radiology), RTT (Radiotherapy Technologist), RO (Radiation Oncologist) and PA (Physician Assistant^4^).

The accuracy of the acquired data relies on the knowledge and diligence of the respondents. Anomalies regarding technical specifications of equipment were checked with the respective vendors afterwards. The following list of vendors have been listed: Vendor C (Canon Medical Systems, Otawara, Tochigi), Vendor G (GE Healthcare, Chicago, IL) Vendor P (Philips, Best, The Netherlands), Vendor S (Siemens Healthineers, Erlangen, Germany) and the following phantoms are mentioned in the text: Model 438 (Gammex, Middleton WI, USA), Model 062 M (CIRS/SunNuclear, Melbourne, FL, USA) and Model 467 (Gammex, Middleton, WI, USA).

## 3 Results

### 3.1 CT scanner specifications and protocol optimization

From the 47 institutions, 31 responses were obtained: a response rate of 66 %. Three institutions chose to provide their answers anonymously. For the identifiable institutions, a balance was observed in Belgian (48 %) vs. Dutch (52 %) institutions as well as in academic (42 %) vs. non-academic (48 %) institutions.

In total, 58 fan-beam CT scanners for RT were in use by the 31 institutions. Specifications were provided for 38 scanners, selected by the respondents for either being the newest scanner, one of more identically configured of the same vendor or the only scanner inside the RT department. For 36/38 of the scanners, the installation date was specified; 81 % was younger than seven years (reference date: Jan 1, 2023).

**Fig. 1:**
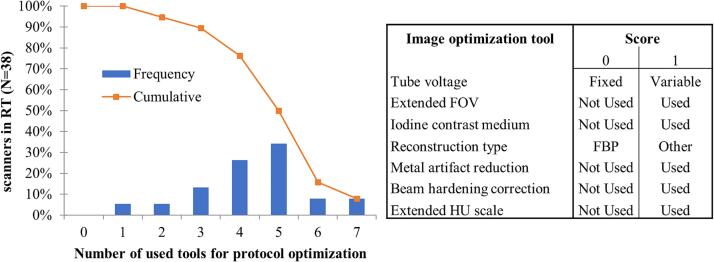
Histogram with inverse cumulative frequency depicting reported clinical use of seven image optimization tools, receiving a binary score: tube voltage (0 score when fixed to 120 kV), extended FOV (0 score when not used), iodine contrast medium (0 score when not used), reconstruction type (0 score when only FBP is used), metal artefact reduction (0 score when not used), beam hardening correction (0 score when not used or respondent being in doubt), extended CT number scale (0 score when not used). In all other cases a value of 1 has been assigned.

The following seven image optimization options for delineation and dose calculation were investigated: tube voltage adjustment, extended field-of-view (FOV), iodine contrast administration, reconstruction methods, metal artifact reduction, beam hardening correction and extended CT number scale. If an option was clinically used, it received a score of 1 (otherwise 0). Four or more of the aforementioned options are used on 76.3 % of the scanners (see Fig. 1). The score did not correlate with scanner age (Spearman's significance 0.34).

On the majority (68 %) of the 38 scanners, images are reported to be acquired with a fixed X-ray tube voltage and with more than 32 slices (53 %) simultaneously. In case of iodine contrast administration, mostly variable volumes are used depending on treatment site, patient characteristics or tube voltage (47 %) or a fixed volume of contrast agent is used (40 %). For image reconstruction, often an iterative image reconstruction algorithm is used in combination with metal artefact reduction, if metal is present. In fewer cases (34 %) beam hardening correction is added to the reconstruction (see Table 1) Whereas for electron treatment no use of dedicated image acquisition or reconstructions is reported, specific slice thicknesses (7/19), lower dose (1/19) or limited FOV (2/19) have been implemented in a minority of the institutions for brachytherapy. The main tasks of CT images for brachytherapy were verification of applicator position (15/19) or LDR seed implants (8/19) and almost never (3/19) to perform heterogeneity-based dose calculation.

**Table 1:** Summary of technical specifications and clinical use for 38/58 of the reported CT scanners. IR = Iterative Reconstruction, FBP = Filtered Back Projection, AI = Artificial Intelligence.

Machine	Who is the vendor of the scanner?	CT Vendor S	48 %
		CT Vendor G	26 %
		CT Vendor P	21 %
		CT Vendor C	5 %
Acquisition	Is a fixed or variable tube voltage used?	Fixed	68 %
		Variable	32 %
	Simultaneously acquired slices?	>32	53 %
		≤32	47 %
	Iodine contrast-enhancement (adults)?	Variable volume	47 %
		Fixed volume	40 %
		Any	13 %
Reconstruction	Which image reconstruction algorithm is used?	IR only	52 %
		IR,FBP	29 %
		FBP	13 %
		IR,AI	3 %
		AI only	3 %
	Is beam hardening correction applied?	No	55 %
		Yes	29 %
		Sometimes	5 %
		Do not know	11 %
	Is metal artefact reduction applied?	Yes, if metal is present	84 %
		Yes, always	8 %
		No	8 %

### 3.2 Dose calculation

**Fig. 2:**
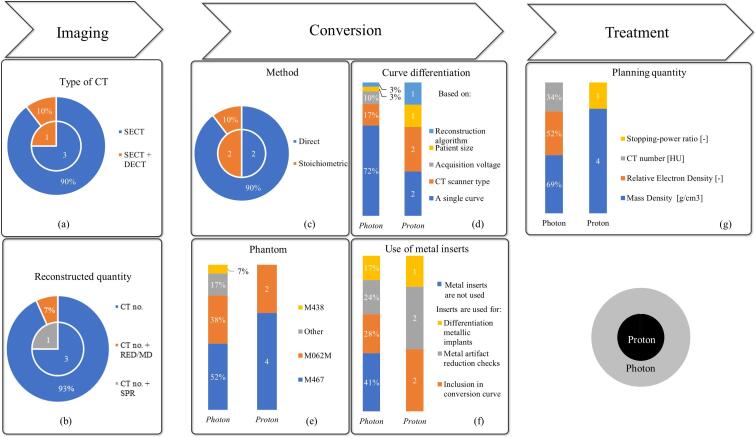
Summary on the survey results on photon (outer ring, N = 29) and proton dose calculation (inner ring, N = 4). Photon related numbers are presented by percentages, while for protons, absolute numbers are given. Where respondents could only select one option, results are graphed as parts of a ring chart. If respondents could select more than one option, the results are graphed as a bar chart, thus sums of percentages can become larger than 100 %. Abbreviations: SECT = Single Energy Computed Tomography, DECT = Dual-Energy Computed Tomography, MD = Mass Density [g/cm^3^], RED = Relative Electron Density [-], SPR = Stopping Power Ratio [-]. (For interpretation of the references to colour in this figure legend, the reader is referred to the web version of this article.)

Answers on Single Energy CT (SECT) for photon, proton, electron, and brachytherapy dose calculation were collected from 29, 4, 19, and 11 institutions, respectively. Fig. 2 illustrates the numbers collected on photon and proton dose calculations: photons in percentages and protons in absolute numbers (always relative to four) separated by a semi-colon. Dual-energy CT (DECT) is used additionally for photon dose calculations in 3 institutions (10 %) – see Table A.1 in the Supplementary Materials – and proton dose calculations in one institution (Fig. 2a). For photon dose calculations, all institutions reconstruct their scans in Hounsfield Units (HU). Two institutions (7 %) additionally reconstruct their scans in CT numbers reflecting relative electron density (RED) or mass density (MD) using an algorithm for direct dose calculation [16]. For proton dose calculations, all institutions reconstruct their CT scans in HU and one institution makes additional reconstructions in Stopping Power Ratio (SPR) [17], [18] (Fig. 2b).

Two main methods are used for setting up the conversion from CT numbers in HU to a quantity that is required by the TPS for dose calculation. Mainly a direct method is used meaning that the CT curve is directly defined by measured CT numbers of plastic tissue-equivalent substitutes (90 % photon institutions, 2 protons institutions). In the second stoichiometric method, the conversion curve is defined by calculated CT numbers of biological tissues determined by measuring CT numbers of tissue substitutes (10 %; 2) [19] (Fig. 2c).

All institutions create their own conversion curves for both photon and proton dose calculations. For electron dose calculations, the photon dose calibration curves are used. For brachytherapy, the majority uses water-based dose calculation and about 26 % of the institutions account for tissue heterogeneity. The majority (72 %; 2) of the institutions uses a single curve for dose calculation. Some institutions define dedicated curves to be able to take scanner type (17 %; 2), X-ray tube voltage (10 %; 0), image reconstruction algorithm (3 %; 1) and patient diameter into account (3 %; 1) (Fig. 2d).

The majority (97 %; 4) of the institutions use commercial phantoms to define their CT conversion curves. The M467 and M062M phantoms are the most popular (Fig. 2e). Apart from the aforementioned tissue substitutes, the phantoms can also contain metal inserts. The latter are used for photon treatment modality conversion curves in more than half of the institutions and serve multiple purposes: for inclusion in the conversion curve (28 %; 2), for metal artefact reduction performance (24 %; 2) and for differentiation of metal alloys of implants (17 %; 1) (Fig. 2f). For dose calculation, different quantities are required by the treatment planning system. Mainly MD and RED are used in photon dose calculation and MD for proton dose calculation (Fig. 2g). The same answers for curve differentiation, phantom use, and metal insert use were given by the institutions that use DECT for photon dose calculation, as when SECT is used.

### 3.3 Quality assurance (QA)

In the participating institutions the QA tasks are allocated between MPE RT (28/31), MPA (23/31), RTT (22/31) and MPE RD (15/31). In most centers (29/31) the QA tasks are divided between two or more professions. In Belgian centers, the MPE RT mainly collaborate with the MPE RD (12/16 in Belgian institutions vs 2/15 in Dutch institutions), whereas in the Netherlands they mainly collaborate with the MPA (8/16 in Belgian institutions vs 12/15 in Dutch institutions). In most institutions (22/31) RTTs are also tasked for to perform part of the QA tasks.

**Fig. 3:**
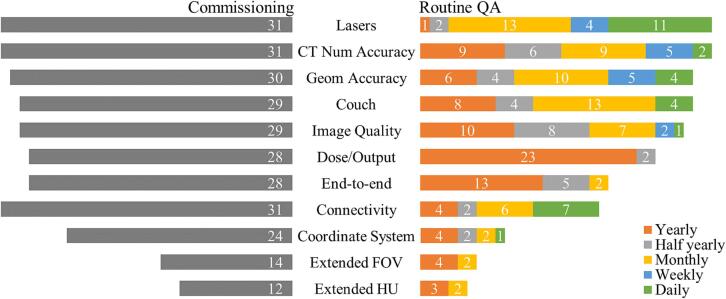
Overview of the CT quality assurance tests performed during commissioning (total centers N = 31) and during periodic time points: yearly, half yearly, monthly, weekly, and daily.

Fig. 3 gives an overview of the CT QA tests performed during commissioning and during regular QA checks at different frequencies. Three main components are evaluated by all centers during commissioning: accuracy and stability of CT numbers, lasers (e.g., stability, distance to isocenter, mobile laser movement) and connectivity and component communication. Most centers (>24/31) additionally check following items during commissioning: geometrical accuracy of reconstructed images, couch stability and movement accuracy, image quality (e.g., high and low contrast resolution, Modulation Transfer Function), CT dosimetry (CT dose index measurements, half-value layer determination), end-to-end testing and coordinate transformations between components. The extended FOV is commissioned in 14 of the in total 31 centers and the extended CT number scale in 12. All tests are also included in routine QA schedule with a frequency varying between different institutions. Most tests, such as dosimetry, are performed on a yearly basis while only a limited amount of QA tasks is performed at higher frequencies such as daily or weekly. Lasers and connectivity are routinely selected for a daily check or weekly check, while dosimetry, end-to-end testing, and image quality are typically performed yearly. Additional information and figures are added to the supplementary materials.

## 4 Discussion

We conducted a survey on the current practice regarding QA for fan beam CT in RT. In this paper, we focused on imaging for dose calculation and delineation. Most institutions reported that CT conversion curves for dose calculation were based on SECT. Even though DECT has been shown to improve range prediction and clinical safety margin reduction in proton therapy [18], [20], its implementation is limited in Belgian and Dutch institutions. An equivalent low number using DECT with photon dose calculations has been reported. However, a workflow for the use of split filter DECT-based photon RT has been recently evaluated to be a feasible alternative to SECT-based photon RT, with possibilities for improved delineation accuracy [21].

Defining CT conversion for dose calculation and deciding which parameters necessitate separate conversion curves is important when commissioning CT scanners for RT. This is reflected by the fact that all institutions constructed their own CT conversion curves and that for some institutions multiple curves have been deemed necessary for scanner type, X-ray tube voltage, reconstruction algorithm or patient diameter. Rather than using multiple conversion curves to improve dose calculation accuracy, most centers go for a robust clinical workflow with only one conversion curve. For the same reason, most institutions chose to use a fixed X-ray tube voltage.

Unfortunately, fully automated selection of conversion curves by the TPSs based on the information available from the DICOM headers, in addition to scanner name is limited to scripting options in the treatment planning system. Thus, solutions for error-safe manual or semi-automated selection of the curves ask considerable effort. Nevertheless, quantitative reconstruction kernels for direct dose calculation yielding quantities independent of the X-ray spectrum such as RED and MD, as opposed to HU [16] open the path to varying X-ray tube voltage selection. Possible reasons for the low number of implementations of such quantitative kernel are: current one-vendor commercial availability, the lack of implementation guidelines, and the lack of image quality evaluation for tissue delineation on this dataset. Perhaps the RT community is now prepared to move beyond a universal CT image-set that serves both delineation and calculation objectives. Separate and dedicated CT image reconstructions could be used for either image delineation or dose calculation.

As the majority of the CT scanners (81 %), dedicated to the radiotherapy workflow, in Belgium and the Netherlands is younger than seven years, modern reconstruction techniques, beam hardening correction (BHC), and metal artefact reduction (MAR) are widely available to create geometrically accurate and artefact-reduced images. Fig. 1 shows that vendor-provided tools are on average well used. It was reported that most institutions use a fixed X-ray tube voltage, limiting the scope for image quality (and dose) optimization of planning CT scans [22].

DECT based image post-processing has proven to enhance image contrast through low keV pseudo-monoenergetic images in comparison to conventional CT [23], [24], [25], [26]. There is still a need for evidence to support the potential improvements in auto-delineation of OAR or reduction of delineation variability [23], [27], [28].

The reported variation in testing frequencies for routine QA may reflect either differences in the frequency of specific tests, in the types of tests performed for the same element, or a combination of both. For example, a quick image quality check is typically part of the daily CT scanner startup routine, whereas a more extensive CT image quality assessment may be performed once or twice a year.

The tests that need to be performed and their frequencies depend on the task for which the CT images are used, and which risks have been identified. Instead of strict frequencies, a shift occurs towards risk assessment-based guidelines on frequency and type of testing. This would fit the goal of producing practical guidelines such that users can design an efficient and thorough QA program tailored to local conditions and usage patterns.

In Belgium and the Netherlands, CT imaging for brachytherapy is applied twice as much for positioning verification of applicators and of seed implants [29], [30] than for dose calculation. In case of the latter use, a water-based environment is predominantly used [31]. By this, a focus on optimizing image quality for tissue and applicator or seed visualization is beneficial to the Dutch and Belgian brachytherapy community. The use of smaller reconstructed FOV and of thinner CT slices has been reported in our survey and rewarded with increased spatial resolution. Previously, guidelines were published that recommended dedicated values for slice thickness to use for either applicator [32] or seed [33] positional accuracies and segmentation. When a seed auto-contouring algorithm is used, the most appropriate slice thickness is to be evaluated against the performance of this algorithm.

To address the increased complexity of determining the optimal image acquisition and reconstruction parameters, respondents have suggested that collaboration with MPEs and medical doctors of the radiology department would be helpful.

In RT, multiple CT acquisitions mostly with extended field diameters and often considerable scan lengths to include relevant OAR for delineation [34] are acquired for different purposes: adaptive offline RT treatments, tailored reconstructions for delineation or for dose calculation. These acquisitions can thus lead to an additional CT radiation dose that should be considered during the protocol optimization. Knowledge of the imaging dose is an important prerequisite for carrying out optimization, and will further increase in importance in proton therapy where accurate positioning of the patient is crucial [35]. The process of optimization should ensure a suitable CT image quality allowing for accurate outlining of the treatment target and surrounding organs whilst minimizing radiation dose received by the patient [22]. Effort has been done to establish dose reference levels for RT treatment planning computed tomography scans for adult patients in the U.K by Woods et al. [34].

To conclude, we conducted an institutional survey to quantify the status, professional challenges, and future directions of QA for fan-beam CT in RT. This work focusses on the two different objectives that affect protocol optimization: dose calculation and tissue delineation. Technical evolutions like DECT for proton dose calculation or a quantitative algorithm for direct photon dose calculation have been implemented only gradually among the respondents. Along all efforts to obtain accurate tissue characterizing quantities (RED, MD, or SPR), a strong emphasis on optimized image quality regarding tissue visualization has been apparent. Novel solutions that guide users safely towards the tissue characterization quantities needed for dose calculation and reduce the burden of image acquisition and reconstruction dependencies can pave the way for decoupling the dual usage of a planning CT. By this, the community could benefit from a larger degree of freedom in protocol optimization for both tasks.

## CRediT authorship contribution statement

**Esther Decabooter:** Conceptualization, Methodology, Formal analysis, Writing – original draft, Writing – review & editing. **Guido C. Hilgers:** Conceptualization, Formal analysis, Writing – original draft, Writing – review & editing. **Joke De Rouck:** Conceptualization, Methodology, Formal analysis. **Koen Salvo:** Conceptualization, Methodology, Formal analysis. **Jacobus Van Wingerden:** Conceptualization. **Hilde Bosmans:** Conceptualization. **Brent van der Heyden:** Conceptualization, Writing – review & editing. **Sima Qamhiyeh:** Conceptualization. **Chrysi Papalazarou:** Conceptualization, Writing – review & editing. **Robert Kaatee:** Conceptualization. **Geert Pittomvils:** Conceptualization, Methodology, Resources. **Evelien Bogaert:** Conceptualization, Methodology, Formal analysis, Writing – review & editing, Supervision.

## Declaration of competing interest

The authors declare that they have no known competing financial interests or personal relationships that could have appeared to influence the work reported in this paper.

## Footnotes

3 BSc.

4 MSc.
