# Supplementary material for: Survey on fan-beam computed tomography for radiotherapy: Imaging for dose calculation and delineation
Source: Phys Imaging Radiat Oncol. 2023 Dec 6;29:100522. doi: 10.1016/j.phro.2023.100522 (PMC10750173; doi:10.1016/j.phro.2023.100522)
Supplement: Supplementary data 1 [file mmc1.pdf]

Appendix A. Supplementary data

A.1 CT scanner specifications and protocol optimization

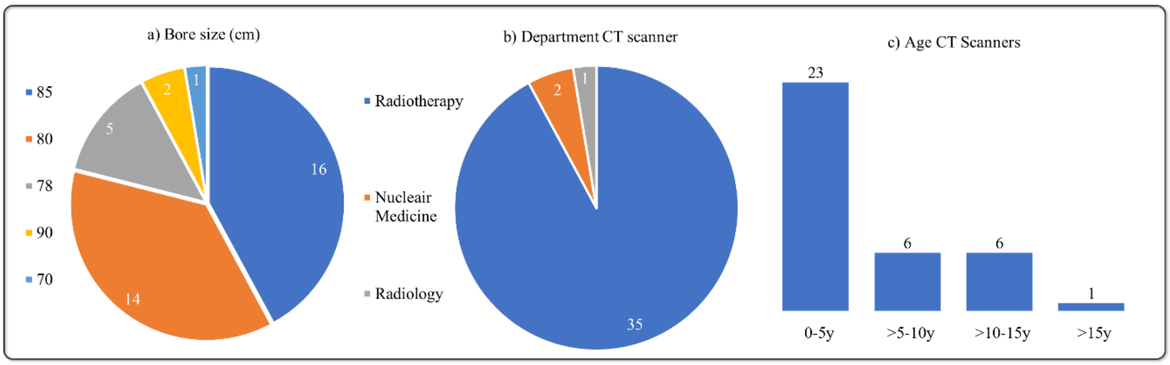

Fig. A.1. Additional information on technical specifications and clinical use for 38/58 of the reported CT scanners: bore size in cm (a), location (b), and age in years (c)

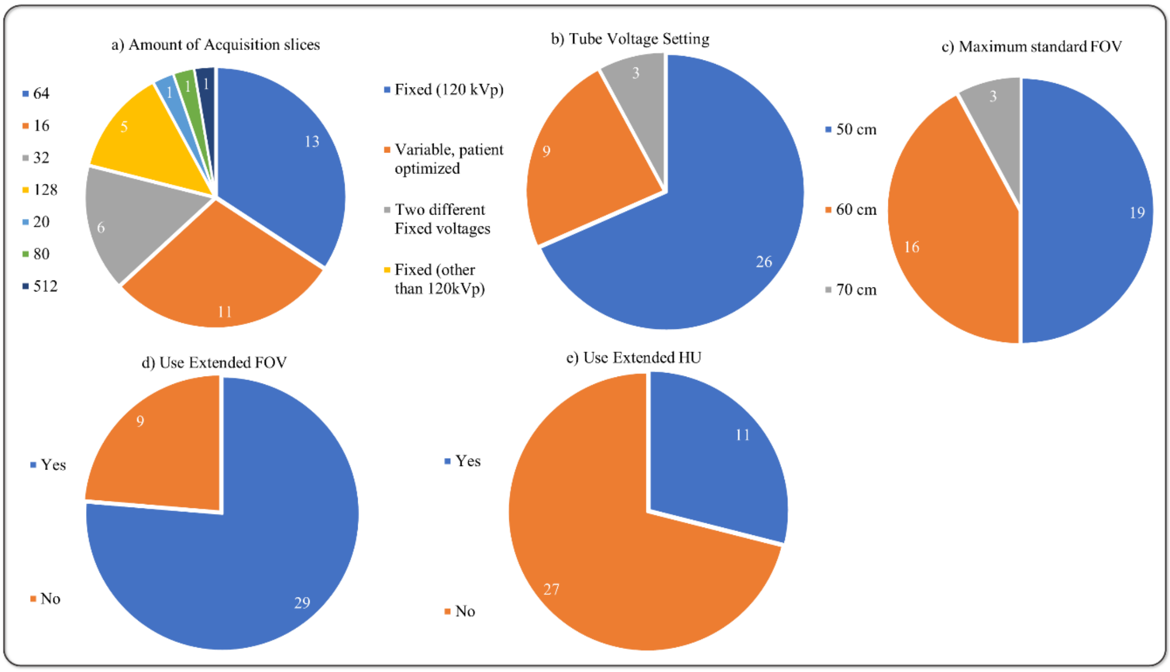

Fig. A.2. Additional information on clinical use for 38/58 of the reported CT scanners: the amount of simultaneously acquired CT slices (a), the tube voltage settings used (b), maximum diameter used as standard FOV (c), number of CT scanners on which extended FOV is used (d), number of CT scanners on which extended HU scale is used (e).

A.2 Dose calculation

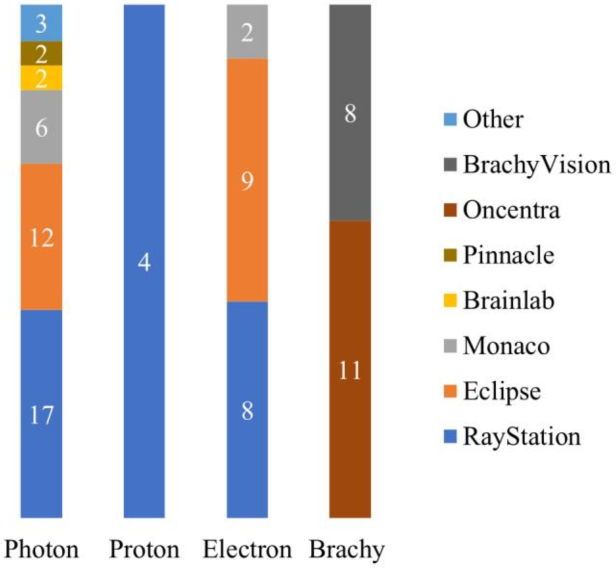

Fig. A.3. Treatment planning systems used per treatment type.

Table A.1 Detailed information for three centers that used dual-energy CT (DECT) additionally for photon dose calculations.

Center	Construction method of Conversion Curve for photon dose calculation
17	Validated the use of their SECT conversion curve on a 120 kVp-like blended DECT image set, reconstructed with a classical kernel. Images originated form a Siemens Somatom Drive (containing two X-ray tubes: dual-source) and a Somatom Confidence (sequential acquisitions: dual-spiral) scanner. Any separate DECT curve was deemed necessary for photon dose calculation.
27	Used a dedicated conversion curve for a 120 kVp-like contrast-enhanced, blended CT reconstruction which originates from a fast kVp switching CT scan technique (General Electric, Revolution CT)
31	Validated the use of their SECT conversion curve on the high energy acquisition (140 kVp) of a Twin Spiral DECT acquisition by a Siemens Go.Open Pro scanner. The conversion curve has been setup for DirectDensityTM (®Siemens Healthineers) reconstructed datasets. Thus the high energy acquisition is also reconstructed with this algorithm when used for photon dose calculation.

A.3 Quality assurance (QA)

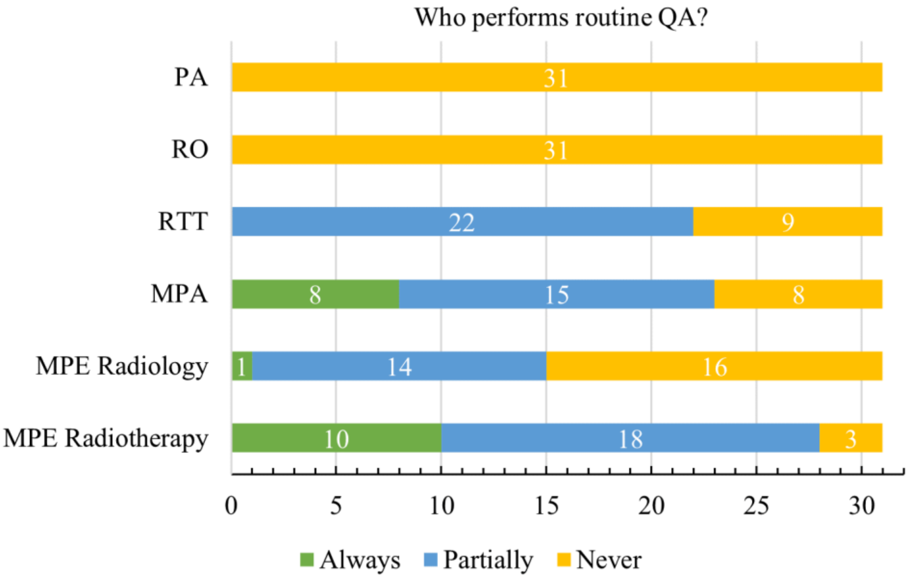

Fig. A.4. Professions performing routine QA at different frequencies. MPA (Medical Physics Assistant), MPE RT (Medical Physics Expert Radiotherapy), MPE RD (Medical Physics Expert Radiology), RTT (Radiotherapy Technologist), RO (Radiation Oncologist) and PA (Physician Assistant).
